# Mortality and use of implantable cardioverter defibrillators in patients with low ejection fraction following non-ST-segment elevation myocardial infarction

**DOI:** 10.1093/europace/euaf036

**Published:** 2025-02-17

**Authors:** Nina Stødkilde-Jørgensen, Kevin Kris Warnakula Olesen, Christine Gyldenkerne, Malene Kærslund Hansen, Roni Ranghoej Nielsen, Jens Cosedis Nielsen, Michael Maeng

**Affiliations:** Department of Cardiology, Aarhus University Hospital, Palle Juul-Jensens Boulevard 99, 8200 Aarhus N, Denmark; Department of Clinical Medicine, Aarhus University, Palle Juul-Jensens Boulevard 99, 8200 Aarhus N, Denmark; Department of Cardiology, Aarhus University Hospital, Palle Juul-Jensens Boulevard 99, 8200 Aarhus N, Denmark; Department of Cardiology, Aarhus University Hospital, Palle Juul-Jensens Boulevard 99, 8200 Aarhus N, Denmark; Department of Clinical Medicine, Aarhus University, Palle Juul-Jensens Boulevard 99, 8200 Aarhus N, Denmark; Department of Cardiology, Aarhus University Hospital, Palle Juul-Jensens Boulevard 99, 8200 Aarhus N, Denmark; Department of Clinical Medicine, Aarhus University, Palle Juul-Jensens Boulevard 99, 8200 Aarhus N, Denmark; Department of Cardiology, Aarhus University Hospital, Palle Juul-Jensens Boulevard 99, 8200 Aarhus N, Denmark; Department of Clinical Medicine, Aarhus University, Palle Juul-Jensens Boulevard 99, 8200 Aarhus N, Denmark; Department of Cardiology, Aarhus University Hospital, Palle Juul-Jensens Boulevard 99, 8200 Aarhus N, Denmark; Department of Clinical Medicine, Aarhus University, Palle Juul-Jensens Boulevard 99, 8200 Aarhus N, Denmark; Department of Cardiology, Aarhus University Hospital, Palle Juul-Jensens Boulevard 99, 8200 Aarhus N, Denmark; Department of Clinical Medicine, Aarhus University, Palle Juul-Jensens Boulevard 99, 8200 Aarhus N, Denmark

**Keywords:** Heart failure, Implantable cardioverter defibrillators, Malignant arrhythmias, Mortality, Guidelines

**What’s new?**

- Patients with non-ST-segment elevation myocardial infarction, obstructive coronary artery disease, and left ventricular ejection fraction ≤35% demonstrated a high 1-year mortality risk and low 1-year implantable cardioverter defibrillator implantation rates.

In patients with non-ST-segment elevation myocardial infarction (NSTEMI), ischaemic events can lead to arrhythmogenic substrates. Further exacerbated by a reduced left ventricular ejection fraction (LVEF), these substrates constitute a risk of sudden cardiac death (SCD). To prevent SCD, European guidelines recommend an implantable cardioverter defibrillator (ICD) in patients with ischaemic cardiomyopathy, symptomatic heart failure, and LVEF ≤35%, after 3 months of optimal medical therapy.^1^

These recommendations are based on pioneering randomized trials demonstrating significant reductions in all-cause mortality after ICD implantation.^2,3^ However, advances in the treatment of NSTEMI and heart failure have improved prognosis since these trials.^1,4^

Applying a subgroup of these patients, we assessed the incidence of all-cause mortality, cardiovascular mortality, and ICD implantation in a Danish all-comers cohort with first-time NSTEMI and LVEF ≤35%.

In this observational cohort study, patients undergoing coronary angiography (CAG) from 2010–2021 were identified from the Western Denmark Heart Registry and cross-linked with nationwide healthcare registries.^5^ Inclusion required first-time NSTEMI, obstructive coronary artery disease (CAD), and LVEF 10–35% as measured just before the index CAG. Exclusion criteria included previous myocardial infarction, percutaneous coronary intervention (PCI), coronary artery bypass grafting (CABG), ICD implantation, or a prior diagnosis of heart failure. Mortality incidence was calculated as 1- and 5-year cumulative incidence proportions (CIP), and the incidence of ICD implantation as 1-year CIP accounting for competing risk of all-cause mortality. The analyses were made using Stata/MP v. 18.

In total, 993 patients with NSTEMI, obstructive CAD and LVEF ≤35% underwent examination with CAG. Of these, 846 (85%) had coronary revascularization performed within 90 days, comprising 600 PCIs and 22 CABGs performed within 3 days, and 95 PCIs and 139 CABGs performed between >3 and 90 days, with some undergoing more than one procedure. Median age was 74 years (Q1–Q3: 66–81 years), and 679 (68%) were men. Within the first year, 200 patients died [CIP 20%; 95% confidence interval (CI) 18–23%], while 397 patients (CIP 44%; 95% CI: 41–48%) died within 5 years (*Figure 1*). Of these, 129 (CIP 13%; 95% CI: 11–15%) and 205 (CIP 22%; 95% CI: 20–25%) were cardiovascular deaths, respectively. Only 50 patients (CIP 5%; 95% CI: 4–7%) received an ICD within the first year: 30 patients received an ICD within the first 3 months, while 20 patients received an ICD between 4 and 12 months (*Table 1*).

**Figure 1:**
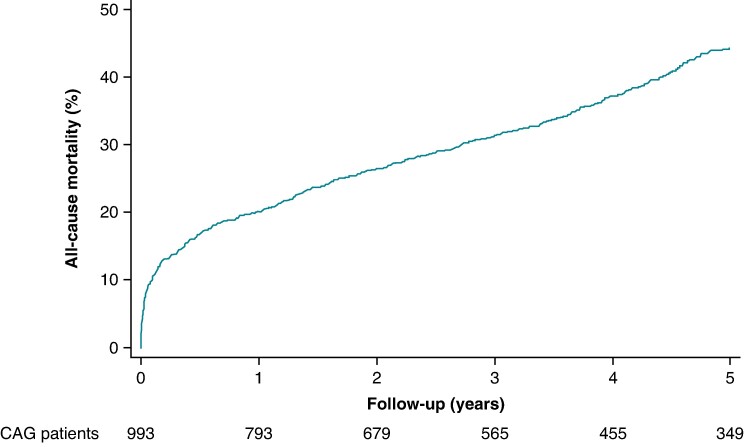
Five-year cumulative incidence of all-cause mortality in NSTEMI patients with LVEF ≤35%. The figure depicts the cumulative incidence of all-cause mortality up to 5 years of follow-up in patients with first-time NSTEMI, obstructive CAD, and LVEF ≤35%. CAD, coronary artery disease; LVEF, left ventricular ejection fraction; NSTEMI, non-ST-segment elevation myocardial infarction.

**Table 1:** Number of ICD implantations per time period after NSTEMI diagnosis

	ICD, *n*
0–3 months	30
4–6 months	7
7–9 months	4
10–12 months	9
Total	50

ICD, implantable cardioverter defibrillator; NSTEMI, non-ST-segment elevation myocardial infarction.

In this cohort with first-time NSTEMI, obstructive CAD, and LVEF ≤35% at the index procedure, we found high 1- and 5-year incidences of all-cause mortality. Moreover, we observed a low 1-year rate of ICD implantations despite guidelines recommending a decision after 3 months of optimal medical therapy. This low implantation rate could have multiple causes: Patients may have been deemed too fragile, with life-expectancy <1 year, LVEF may have recovered to ≥35% during the up-titration of medical therapy, they may have become asymptomatic after optimal medical therapy, or maybe too few patients were referred. Still, we find that the strikingly low incidence of ICD implantation is of concern, particularly considering that up to ∼45% of patients with myocardial infarction do not improve their LVEF afterwards, and such patients with no recovery are at increased risk of death.^6^ Denmark holds one of the highest implantation rates in Europe,^7^ making it highly unlikely to be an issue restricted to Western Denmark.

In conclusion, in a high-risk cohort of patients with first-time NSTEMI and documented obstructive CAD examined in a country with a high implantation rate of ICDs, we found a surprisingly low rate of ICD implantation in the first year following index MI. Further studies are warranted to assess whether the indication for ICD implantation remains after 3 months of optimal medical therapy and to evaluate the associated risk of non-arrhythmic death.^8^

## Ethical considerations

The study was approved by a regional branch of the Danish Data Protection Agency (record no. 1-16-02-193-18). Patient consent requirements were waivered upon approval by regional data protection authorities (record no. 14-45-70-24-22).

## Data Availability

Due to patient confidentiality restrictions, the data supporting the findings of the study are not publicly available. Further enquiries can be directed to the corresponding author.
